# Human land‐use changes the diets of sympatric native and invasive mammal species

**DOI:** 10.1002/ece3.10800

**Published:** 2023-12-06

**Authors:** Antje Chiu‐Werner, Menna Jones

**Affiliations:** ^1^ School of Natural Sciences University of Tasmania Hobart Tasmania Australia

**Keywords:** diet, eDNA, human activities, invasive species, land‐use, native species, stable isotopes

## Abstract

The consequences of biological invasions and habitat degradation for native biodiversity depend on how species cope with the individual and synergetic challenges these processes present. To assess the impact of anthropogenic land‐use on the food web architecture of an invaded community, we examine the diets of nine native and two highly invasive mammal species at different trophic levels, inhabiting different land‐uses across six biogeographic regions in Tasmania, Australia. We use two complementary methods, environmental DNA metabarcoding analysis (eDNA) of faeces and stable isotope analysis (SIA) of nitrogen (N) and carbon (C) in whole blood, to account for the high interindividual and temporal variability in the diets of multiple species simultaneously. eDNA showed regionalisation in the diet of smaller species, with land‐use further defining dietary taxa within each region. SIA revealed that bioregion and land‐use influence the δ^13^C values of all carnivore species and omnivores, whereas the δ^15^N values of these species are influenced only by land‐use and not bioregion. Including multiple species showed that native rats are changing their diet in response to the presence of invasive rats, an impact that would have otherwise been attributed to land‐use. Our findings demonstrate that human activities and invasive species are moulding the diets of invaded communities, raising questions about the potential impacts that dietary modifications will have on the life‐history traits and the evolutionary consequences these modifications might have on the survival of native species. This highlights the urgency of including human activities in ecological studies and the importance of targeting multispecies assemblages to gain a better understanding of synergetic impacts on native biodiversity.

## Graphical abstract

We simultaneously study the diet of nine native and two invasive mammal species inhabiting three different anthropogenic land‐uses across six bioclimatic regions using environmental DNA and stable isotope analysis. We show that land‐use influences the trophic relationships in invaded communities. Our study highlights the importance of including human activities in research design and studying multispecies assemblages to discern between the effects of invasive species and anthropogenic land‐use.

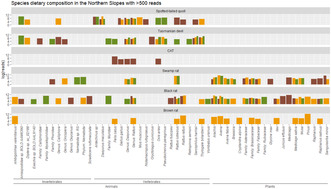

## Resumen

Las consecuencias de invasiones biológicas y degradación de hábitat en la biodiversidad nativa de un lugar dependen de las respuestas de las especies a los desafíos individuales y conjuntos que estos procesos les presentan. En este estudio examinamos la dieta de nueve mamíferos nativos y dos mamíferos altamente invasivos, pertenecientes a diferentes niveles tróficos, y que habitan diferentes usos de suelo en seis regiones bioclimáticas en Tasmania, Australia con el fin de examinar el impacto de diferentes usos de suelo en la estructura de una red trófica en una comunidad invadida. Para ello utilizamos dos métodos complementarios: ADN ambiental (eDNA) en heces y análisis de isótopos estables (SIA) de nitrógeno (N) y carbono (C) en sangre para dar cuenta, respectivamente, de la alta variabilidad interindividual y temporal en la dieta de estos animales. Los resultados de eDNA mostraron una regionalización en la dieta de las especies más pequeñas, y que el uso de suelo define aún más los taxa dietéticos dentro de cada región. El SIA reveló que los valores de δ^13^C de todas las especies de carnívoros y omnívoros están influenciadas por biorregiones y uso de suelo, mientras que los valores de δ^15^N de carnívoros y omnívoros sólo son influenciadas por el uso de suelo. Nuestros hallazgos demuestran que las actividades humanas y las especies invasoras están moldeando las dietas en comunidades invadidas generando preguntas sobre los potenciales impactos que las modificaciones dietéticas puedan tener sobre las características de las historias de vida de especies nativas y las consecuencias evolutivas que a su vez estas puedan tener en la sobrevivencia de estas especies. Así, este estudio demuestra la urgencia de incluir actividades humanas en los estudios ecológicos y la importancia de enfocarse en ensamblajes de múltiples especies para obtener un mejor entendimiento de los impactos sinérgicos sobre la biodiversidad nativa.

## 1 INTRODUCTION

Habitat loss and biological invasions are among the major drivers of biodiversity change and native species decline (Harfoot et al., 2021), with invasive mammalian predators being the most damaging group for global biodiversity (Doherty et al., 2016). Yet in the research that informs management, the influence of anthropogenic transformation of the landscapes where alien and invasive predators are abundant is often ignored (Elmhagen & Rushton, 2007; Morgan et al., 2017), even though it could be facilitating and intensifying their impact (e.g. Catling & Burt, 1994; Hernandez‐Santin et al., 2016; Hohnen et al., 2016; Wang et al., 2021).

Species coexistence is the outcome of co‐evolutionary processes that reduce competition (Lankau, 2011). Reduction in competition is enabled by resource partitioning (space, time and food) and specialisation (Finke & Snyder, 2008) or by the presence of a third species that can modulate competition (Abrams & Rueffler, 2009). Human land‐use changes the ecological interactions between species, which in invaded ecosystems is often at the cost of native biodiversity (e.g. Norbury et al., 2013; Wang et al., 2021). Changes in interaction strengths alter evolutionary selection pressures on both native and invasive animals, creating eco‐evolutionary feedbacks that are reflected in food web structure (David et al., 2017; Layman et al., 2007; Wainright et al., 2021).

We used the mammal community in Tasmania (Australia) as a case study to assess the impact of anthropogenic land‐use on the architecture of food webs in an invaded community. Tasmania has a relatively uniformly distributed community of mammal species (Chiu‐Werner A., Montalvo‐Mancheno C.S., Driessen M., Jones M.E., unpublished data; Montalvo‐Mancheno et al., 2021), but the abundance of each species varies geographically. For instance, the disease‐induced decline of populations of the top predator and scavenger, the Tasmanian devil (*Sarcophilus harrisii*), has triggered a rise in feral cat (*Felis catus*) numbers through reduced competition and increased presence of carrion in the landscape (Cunningham et al., 2018, 2020). This increase in feral cat numbers is resulting in declines in small‐medium native mammalian prey such as bandicoots (Order Peramelemorphia; Cunningham et al., 2020) and reducing or limiting smaller native predators such as eastern quolls (*Dasyurus viverrinus*) (Fancourt et al., 2015; Hollings et al., 2014, 2016). This effect is exacerbated in agricultural landscapes where feral cat densities are extremely high, potentially supported by abundant invasive prey (rabbits and rodents) (Hamer et al., 2021; Hollings et al., 2014). Tasmania also provides an ideal setting to test the effects of land‐use, as ~49% of the island is protected area (i.e. control), ~23% is managed by forestry and ~21% is agricultural land (Australian Bureau of Agricultural and Resource Economics and Sciences [ABARES], 2022).

We use a combination of environmental DNA metabarcoding analysis (eDNA hereafter; Taberlet et al., 2012) of faeces and stable isotope analysis (SIA) of nitrogen (N) and carbon (C) in whole blood to assess the effects of bioregion (Peters & Thackway, 1998) and anthropogenic land‐uses on the trophic structure of the mammal community in Tasmania. By combining these approaches, we make use of their complementary time periods, allowing us to account for the high interindividual and temporal variability in animal diets. eDNA analysed from faecal samples will represent a snapshot of a single meal, although a large prey item will appear as having low heterogeneity. eDNA allows for the taxonomic characterisation of the DNA present in complex samples such as water, soil and faeces (Bohmann et al., 2014), including the potential to detect underrepresented and soft‐bodied prey species.

SIA provides an integrated picture of the diet of an animal over a specific timeframe. The timeframe represented by SIA is given by the rate at which isotopes are incorporated from the prey into the consumer tissues (turnover rate), which depends on the analysed tissue (Hobson & Clark, 1992). More metabolically active tissues (e.g. internal organs and blood plasma) show faster turnover rates than less metabolically active tissues such as bones or muscle (Boecklen et al., 2011). The usefulness of SIA in ecology relies on the fact that the isotopic signature of consumers reflects that of their diet (Deniro & Epstein, 1976), and it allows to describe a trophic space often used as a proxy for trophic niche size (Newsome et al., 2007). Trophic niches are frequently built using the ratio of stable nitrogen isotopes (^15^N:^14^N; δ^15^N), which will increase by 3–5‰ with each trophic level and can therefore be used as a tool to determine dietary shifts (Hobson & Wassenaar, 2008), and the ratio of stable carbon isotopes (^13^C:^12^C; δ^13^C), which remain relatively stable with trophic level but are heavily influenced by the photosynthetic pathway of the primary producers at the base of the food web (Fry, 2006).

By combining the results of these two different but complementary approaches to studying the diet of multiple species living in sympatry, we expect to obtain a more comprehensive picture of the influence of human land‐use on the diet and trophic relationships of an invaded community. Land‐use alters vegetation composition, which should be reflected in the diet composition of mammal species and potentially in shifts in the breadth of isotopic niches as animals utilise the available food resources. We expect greater overlaps in diet between native and invasive species in human‐modified (plantations and agricultural) land‐uses relative to undisturbed land‐uses because human activities can homogenise landscapes, removing the natural variability of vegetation communities, with similar outcomes in the diversity (richness but particularly abundance) of the animal community inhabiting them.

## 2 MATERIALS AND METHODS

### 2.1 Study site

**FIGURE 1:**
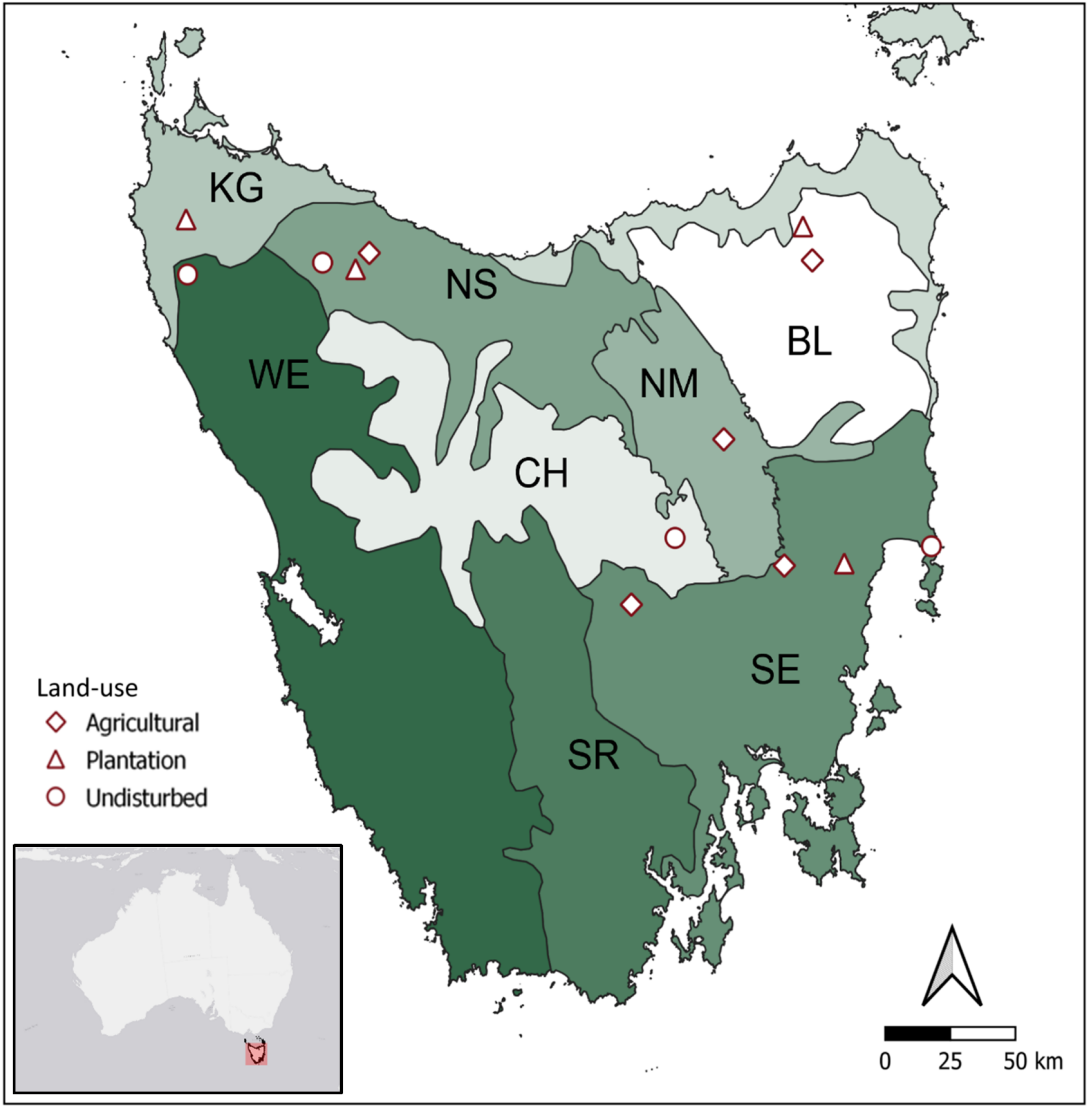
Sample collection sites. Shading distinguishes the bioregions in Tasmania. BL, Ben Lomond; CH, Central Highlands; KG, King; NM, Northern Midlands; NS, Northern Slopes; SE, South East; SR, Southern Ranges; WE, West.

We established 13 study sites of approximately 25 km^2^ each across six different bioregions in Tasmania (Figure 1; Peters & Thackway, 1998). Bioregions are classifications reflecting the underlying spatial organisation of biota in a biogeographic framework (Thackway & Cresswell, 1995). Within each bioregion, we selected landscapes in each of three land‐use categories where they exist (not all landscapes occur in each bioregion): We defined each of the 13 sites using the following criteria:

1. ‘Undisturbed’ landscape: Represents minimal anthropogenic disturbance and is considered the baseline for comparisons in this study. Located within a national park, at least 80% of the area is covered by undisturbed native vegetation (*n* = 4).
2. ‘Plantations’ landscape: Despite being monocultures, we consider ‘Plantations' to represent an intermediate level of disturbance between ‘Undisturbed’ and ‘Agricultural’ because they retain stream‐side reserves and areas of native vegetation for non‐wood values such as threatened species and cultural heritage (Forest Practices Authority, 2015). To match this category, the landscape had to be at least 60% covered by mature (>15 years) pine or eucalypt plantation (usually in a combined matrix with native forests; *n* = 4). These landscapes are rich in edges in the form of roads. For safety reasons, we did not sample forestry sites where harvesting or thinning was taking place.
3. ‘Agricultural’ landscape: This land‐use category was considered to be the most disturbed despite sampling in vegetation remnants (native or plantations). These remnants were located within a landscape that has at least 80% of its area covered by agricultural or farming land (*n* = 5). Remnants represent highly fragmented native habitat and are usually a combination of nature reserves and conservation covenants. The land surrounding them is intensively used for livestock grazing (sheep and cows).

### 2.2 Species and sample collection

We deployed an average of 60 baited traps (±5; 10 dedicated carnivore pipe traps, 10 medium and 20 small‐medium wire cage traps and 20 Elliot (Sherman) traps) during five consecutive nights at each site during the austral winter of 2021, when we expect human land alteration to have the greatest influence on mammals. We baited carnivore and the medium wire cage traps with lamb (*Ovis aries*) and chicken (*Gallus gallus*). Small wire cage traps and Elliot traps were baited with a mixture of rolled oats (*Avena sativa*), peanut butter (*Arachis hypogaea*) and sugar cane (*Saccharum officinarum*) golden syrup. Traps were situated in sheltered locations, not more than 50 m from a road or path and covered with hessian bags. An additional piece of hessian bag and sugar cane mulch were placed inside each trap to provide bedding material for trapped animals. We checked traps starting at dawn. All the trapped feral cats were euthanised.

The eDNA metabarcoding analysis was conducted on all carnivore species: the Tasmanian devil, spotted‐tailed quoll (*Dasyurus maculatus*), eastern quoll (*D. viverrinus*), feral cat and two rodents—the native swamp rat (*Rattus lutreolus*) and the invasive black rat (*R. rattus*)—to address dietary overlap between native and invasive species. For the SIA, we targeted all carnivores and an omnivore: the brushtail possum (*Trichosurus vulpecula*). We opportunistically sampled two more omnivores, the eastern barred and southern brown bandicoots (*Perameles gunii* and *Isoodon obesulus*, respectively), and two fungivores, the long‐nosed potoroo (*Potorous tridactylus*) and eastern bettong (*Bettongia gaimardi*).

#### 2.2.1 DNA metabarcoding

When present in the traps, a small portion of faecal matter was collected in a clean 2 mL tube directly from each trap, and only on the first capture for each individual within the field trip. Upon collection, each sample was placed in a cooler on ice and transferred to a portable −80°C freezer until analysis. We pooled samples for analysis, for each species per site, to reduce the bias found in eDNA analysis towards detecting mainly items present in a recent meal.

#### 2.2.2 Stable isotope analysis

For each trapped adult animal (excluding rodents for which we did not take blood samples), we collected between 0.05 and 0.1 mL of blood by venal puncture of the peripheral ear vein with a 25‐gauge sterile needle and collected the blood in a 1.5 mL clear, uncoated polypropylene tube with leak‐proof o‐ring screw caps. We closed the tube for transportation to the field base, where later the same day we commenced desiccation of the samples by placing the opened tubes on a rack in a sealed container with silica gel (SiO_2_) for 14–21 days. We then closed the tubes until further analysis in the lab. We analysed whole blood as this would reflect the diet consumed by each individual in the 3–4 weeks prior to blood collection, a longer period than for plasma (Klaassen et al., 2010; MacAvoy et al., 2006; Vander Zanden et al., 2015), reducing potential variability in our samples due to seasonality. Blood samples of feral cats were collected immediately after euthanasia, which was done following the guidelines of Tasmania's Cat Management Act 2009.

The study was undertaken with the approval of the University of Tasmania Animal Ethics Committee (permit A0018012), the Department of Primary Industries, Parks, Water and the Environment (permits TFA20079 and TFA21035), Sustainable Timber Tasmania (permit FAA1829) and Reliance Forest Fibre (permit RFF052).

### 2.3 Laboratory analysis

#### 2.3.1 DNA metabarcoding

DNA extraction and metabarcoding were performed at the laboratories of EnviroDNA (Melbourne, Australia) following the processes described in McColl‐Gausden et al. (2021), with the following modifications:

1. We used Qiagen PowerSoil HT kits for extraction on a QiaCube HT system, subsampling approximately 150–200 mg of sample from each pool that had been homogenised using a Qiagen TissueLyser. Each pool contained no more than five individual faecal samples. Hence, for those species for which more than five samples were available per site, these samples were split into two or more pools. The DNA from these pools was then further pooled for library preparation.
2. We used the 12S vertebrate amplicon listed in McColl‐Gausden et al. (2021), the COI amplicon from Zeale et al. (2011) and the trnL P6 loop amplicon from primers *c* and *h* in table 1 of Taberlet et al. (2007).
3. We did not use heterogeneity spacers or SequalPrep normalization (McColl‐Gausden et al., 2021). Instead, we pooled two PCR replicates per sample, and we sequenced these libraries on an iSeq‐100. Otherwise, PCR and library prep for all three amplicons were as described by McColl‐Gausden et al. (2021).

All libraries were sequenced using Illumina iSeq, and the resulting sequences were matched to reference sequence databases (NCBI, 2022) and validated through records on the Atlas of Living Australia (ALA; www.ala.org.au. Accessed 10 August 2022). The markers varied in taxonomic resolution and accuracy, indicating that reference sequences of many Tasmanian species are missing in DNA sequence databases. If the markers indicated the presence of a taxon known not to occur in Tasmania, we assigned the lowest level of taxonomic resolution known to be present in Tasmania for that taxon. Anything below 1% abundance of the total sample was excluded from analysis.

#### 2.3.2 Stable isotope analysis

We pulverised each dry sample using a non‐porous 5 cm agate mortar and pestle to homogenise the sample. We then weighed 0.50 mg into tin capsules and analysed the carbon and nitrogen stable isotopes using flash combustion isotope ratio mass spectrometry (varioPYRO cube coupled to Isoprime100 mass spectrometer) at the Central Science Laboratory of the University of Tasmania, Australia. Stable isotope abundances of nitrogen and carbon are reported in delta (*δ*) values as deviations from their respective standards (air and PDB (Pee Dee Belemnite), respectively) in parts per mil (‰), following the equation:

$$\mathrm{\delta X}\;\left(‰\right)=\left[\left(R_{\text{sample}}/R_{\text{standard}}–1\right)\times 1000\right]$$

where *X* = ^13^C or ^15^N and *R* = the ratio ^13^C/^12^C or ^15^N/^14^N.

Analytical precision was obtained by repetitive measurements of at least three international standards (for nitrogen: IAEA‐N1, IAEA‐N2, USGS40, USGS41 and USGS‐25; for carbon: NBS 21, USGS24, ANU‐NAR‐76H, USGS40 and USGS41) and determined at around 0.1‰ for carbon and nitrogen. The precision for both element contents is 0.1%, and the intrasample variation resulting from homogenisation was <0.02‰.

### 2.4 Replication statement

**Table:** 

(Type of analysis) Scale of inference	Scale at which the factor of interest is applied	Number of replicates at the appropriate scale
(eDNA) Species	Pooled site (Land‐use*Bioregion)	10 Tasmanian devil
		9 Spotted‐tailed quoll
		3 Eastern quoll
		2 Feral cat
		5 Swamp rat
		5 Black rat
		1 Brown rat
(SIA) Species	Land‐use, Bioregion	61 Tasmanian devil
		20 Spotted‐tailed quoll
		28 Eastern quoll
		4 Feral cat
		34 Brushtail possum
		2 Long‐nosed potoroo
		2 Eastern bettong
		8 Southern brown bandicoot
		2 Eastern barred bandicoot

### 2.5 Statistical analyses

#### 2.5.1 eDNA analysis

We used hierarchical cluster analyses (HCA) on the prey items to assess how the diet of each species differed between land‐uses and bioregions, and to uncover patterns in diet data that are consistent with interspecific competition. To determine the optimum number of clusters for each analysis, we used the average silhouette method of the function *fviz_nbclust* in the R package *factoextra* (Kassambara & Mundt, 2020). Dissimilarity values were computed the using Bray–Curtis dissimilarity index in *vegan* (Oksanen et al., 2022), and an agglomerative hierarchical clustering was performed using Ward's minimum variance in *stats* (R Development Core Team, 2022). We then scaled the data using the R function *scale* (R Development Core Team, 2022) to obtain the cumulative variance explained in the dataset using the *fviz_cluster* function of the *factoextra* package (Kassambara & Mundt, 2020). The *fviz_cluster* function reduces the dimensions of the initial set of variables into two principal components (PC1 and PC2) through a principal component analysis (PCA). The R package *cluster* (Maechler et al., 2022) provided the clustering algorithms.

We measured Simpson's index of diversity (1–D) for each species per land‐use to compare them to the width of their respective isotopic signatures when available and to the diets of the species or land‐use in the same cluster. To do so, host DNA reads for each sample were removed *apriori*. When reporting the results of omnivores, if *Avena* and *Arachis* (i.e. from rolled oats and peanut butter, respectively) were present together in a sample, we assume that these originated from the bait and will only be reported in the graphs.

#### 2.5.2 Stable isotope analysis

We performed a qualitative comparison for the isotopic data of each species per land‐use within each bioregion because the sample size per species, per site, was generally <8 (Pearson & Grove, 2013). The same qualitative assessment was conducted to compare the isotopic signature and ranges among sympatric species.

## 3 RESULTS

We collected a total of 113 individual faecal samples and 161 blood samples. Faecal samples resulted in 10 pools for Tasmanian devil samples: nine for spotted‐tailed quolls, three for eastern quolls, two for feral cats, five for swamp rats, five for black rats and one for brown rats (Table 1). Blood samples were analysed individually.

**TABLE 1:** Details on the dietary diversity for each species per land‐use (AG = agricultural, PL = plantation, UN = undisturbed) and per bioregion.

Bioregion	Species	Land‐use	*n* (eDNA /SIA)	Simpson index of diversity (1–*D*)	δ^13^C (‰) mean ± SD	δ^15^N (‰) mean ± SD
Ben Lomond	Eastern quoll	AG	1/2	0.67	−25.51 ± 0.00	8.23 ± 0.57
		PL	3/3	0.58	−23.51 ± 0.08	6.72 ± 0.22
	Spotted‐tailed quoll	AG	1/2	0.81	−25.76 ± 0.81	7.07 ± 0.28
		PL	1/1	0.54	−24.75	10.05
	Tasmanian devil	PL	2/2	0.58	−25.54 ± 0.66	7.434 ± 0.49
	Black Rat	AG	2/−	0.63	*–*	–
		PL	3/−	0.043	*–*	*–*
	*Cat*	*AG*	*−/1*	*–*	*−23.79*	*5.86*
	*Brushtail possum*	*AG*	*−/7*	*–*	*−26.72 ± 0.35*	*3.06* ± 2.07
		*PL*	*−/2*	*–*	*−26.36 ± 0.52*	*6.03 ± 0.21*
	*Eastern barred bandicoot*	*AG*	*−/1*	–	*−25.06*	*4.74*
		*PL*	*−/1*	–	*−26.62*	*7.71*
	*Southern brown bandicoot*	*AG*	*−/1*	–	*−24.76*	*5.65*
Central Highlands	Eastern quoll	UN	13/23	0.75	−25.44 ± 0.29	7.03 ± 0.32
	Spotted‐tailed quoll	AG	3/3	0.87	−25.51 ± 0.10	7.16 ± 0.52
	Tasmanian devil	UN	2/5	0.59	−25.83 ± 0.13	6.41 ± 0.28
		AG	4/8	0.67	−25.49 ± 0.23	7.38 ± 0.64
	*Brushtail possum*	*UN*	*−/5*	–	*−26.01 ± 0.27*	*4.90 ± 0.48*
		*AG*	*−/4*	–	*−25.90 ± 0.35*	*4.07 ± 0.15*
King	Tasmanian devil	UN	6/6	0.75	−25.04 ± 0.50	6.19 ± 0.43
		PL	7/8	0.52	−24.99 ± 0.44	5.72 ± 0.61
	Swamp Rat	UN	1/−	0.57	–	–
		PL	1/−	0.91	–	–
	*Southern brown bandicoot*	*PL*	*−/2*	*–*	*−25.83 ± 0.30*	*4.48 ± 0.26*
Northern Midlands	Spotted‐tailed quoll	AG	4/4	0.11	−24.89 ± 0.18	8.47 ± 1.22
	Cat	AG	1/1	0.79	−24.61	6.43
	*Brushtail possum*	*AG*	*−/5*	*–*	*−25.09 ± 0.85*	*4.73 ± 2.23*
	*Southern brown bandicoot*	*AG*	*−/1*	*–*	*−24.15*	*3.98*
Northern Slopes	Spotted‐tailed quoll	UN	4/4	0.72	−24.47 ± 0.17	6.28 ± 0.69
		PL	1/−	0.78	–	–
		AG	1/−	0.77	–	–
	Tasmanian devil	UN	11/13	0.74	−25.56 ± 0.69	6.97 ± 0.58
		PL	3/3	0.76	−25.81 ± 0.27	6.97 ± 0.19
		AG	5/5	0.75	−26.05 ± 0.28	7.19 ± 0.43
	Cat	AG	1/2	0.86	−24.87 ± 0.53	7.30 ± 0.00
	Swamp rat	UN	2/−	0.72	–	–
		PL	6/−	0.57	–	–
		AG	2/−	0.44	–	–
	Brown rat	PL	1/−	0.41	–	–
	Black rat	UN	2/−	0.81	–	–
		PL	5/−	0.79	–	–
		AG	5/−	0.74	–	–
	*Brushtail possum*	*UN*	*−/1*	–	*−23.86*	*2.35*
		*PL*	*−/2*	–	*−26.32 ± 0.33*	*4.32 ± 0.83*
		*AG*	*−/1*	–	*−25.80*	*3.89*
	*Long‐nosed potoroo*	AG	−/2	–	*−24.49 ± 0.28*	*9.18 ± 0.32*
	*Southern brown bandicoot*	AG	−/4	–	*−26.14 ± 0.75*	*8.47 ± 1.08*
South East	Spotted‐tailed quoll	UN	2/4	0.82	−23.26 ± 1.04	7.63 ± 0.81
		PL	1/1	0.81	−24.83	6.69
		*AG*	*−/1*	*–*	*−24.64*	*7.78*
	Tasmanian devil	UN	5/9	0.74	−23.89 ± 0.51	8.41 ± 0.52
		PL	1/1	0.50	−24.88	7.34
		*AG*	*−/1*	–	*−24.83*	*9.12*
	*Brushtail possum*	*UN*	*−/4*	–	*−26.35 ± 0.29*	*11.24 ± 5.63*
		*PL*	*−/1*	–	*−27.45*	*2.82*
		*AG*	*−/2*	–	*−24.9 ± 0.93*	*4.40 ± 2.92*
	*Eastern bettong*	*PL*	*−/1*	–	*−25.29*	*15.02*
		*AG*	*−/1*	–	*−23.78*	*13.12*

*Note*: ‘*n*’ denotes the number of individuals sampled for eDNA and SIA analyses, respectively. The species in italics were not part of the cluster analysis because no eDNA sample was available for analysis.

### 3.1 eDNA analysis

**FIGURE 2:**
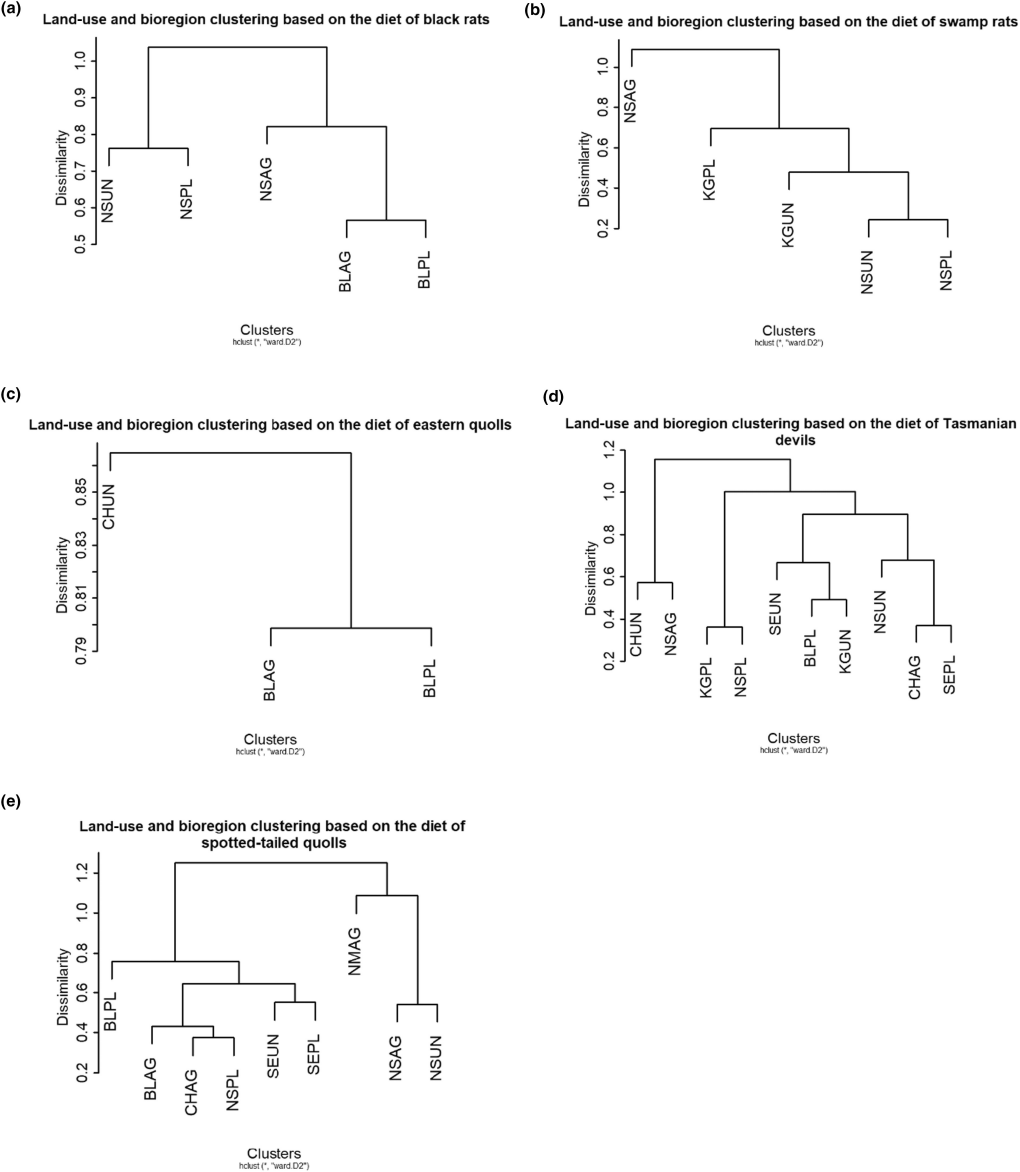
Effects of bioregion (first two letters of each element; BL, Ben Lomond; CH, Central Highlands; KG, King; NM, Northern Midlands; NS, Northern Slopes; SE, South East) and land‐use (last two letters of each element; AG, agricultural; PL, plantation; UN, undisturbed) on the dietary composition of each species (a. black rat, b. swamp rat, c. eastern quoll, d. Tasmanian devil and e. spotted‐tailed quoll) as shown by a hierarchical cluster analysis.

**FIGURE 3:**
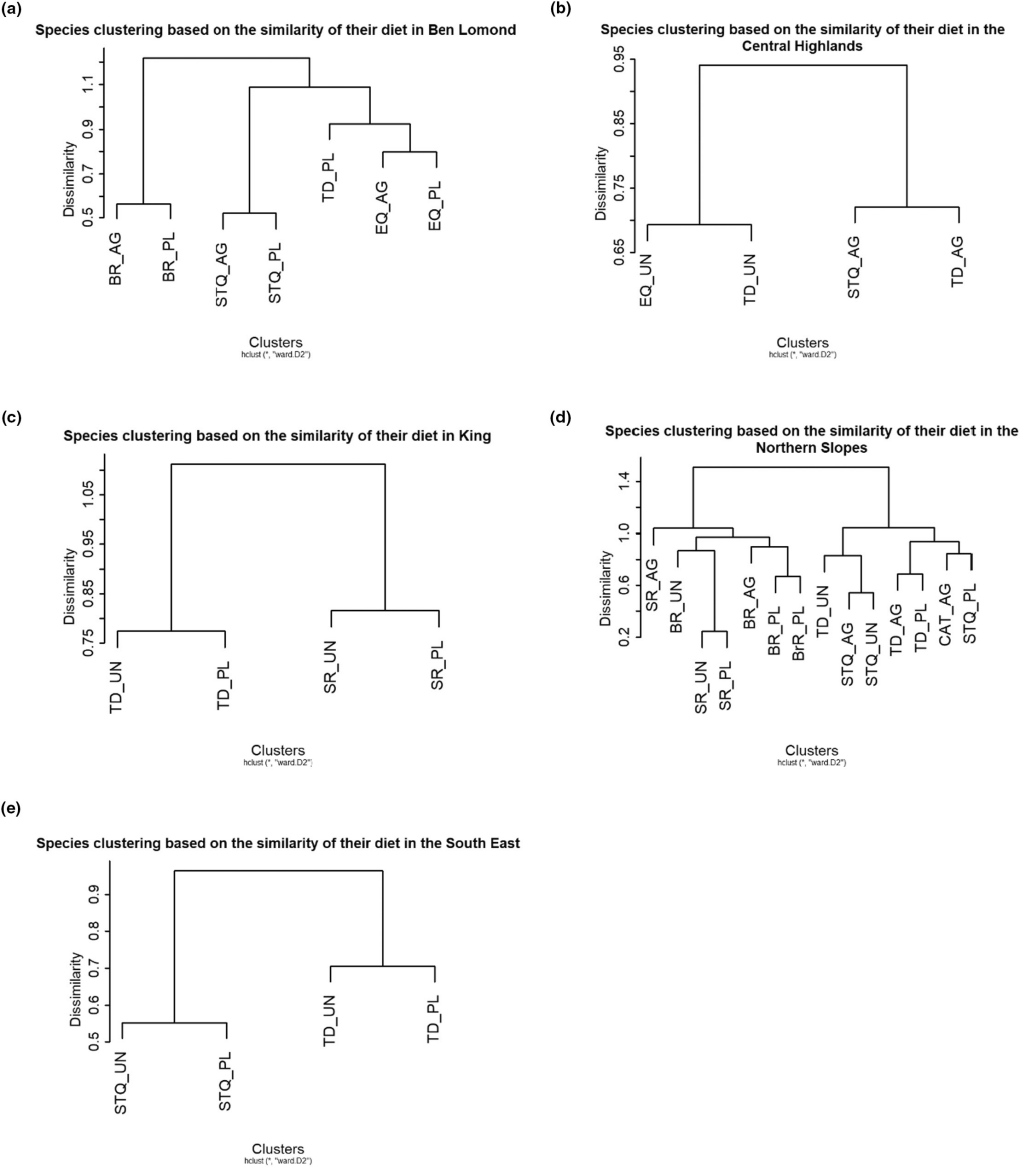
Hierarchical cluster analysis of species per land‐use and bioregion based on the similarity of their diet. (a) Ben Lomond; (b) Central Highlands; (c) King; (d) Northern Slopes; and (e) South East. No cluster analysis was performed for the Northern Midlands because it contained only agricultural landscapes and two species. AG, agricultural landscapes; BR, black rat; EQ, eastern quoll; PL, plantation landscapes; SR, swamp rat; STQ, spotted‐tailed quoll; TD, Tasmanian devil; UN, undisturbed landscapes.

Clustering the dietary taxa found in the faecal samples per species showed a bioregional specialisation in the diet of the smaller species: black rats (Figure 2a; PC1: 69.7%, PC2: 13.2%), swamp rats (Figure 2b; PC1: 78.7%, PC2: 9.9%) and eastern quolls (Figure 2c; PC1: 88.9%, PC2: 11.1%), but not in the larger carnivores: Tasmanian devils (Figure 2d; PC1: 71.8%, PC2: 7.4%) or spotted‐tailed quolls (Figure 2e; PC1: 76.2%, PC2: 6.6%). Clustering species per land‐use and bioregion based on the similarity of their diet showed that in some bioregions (Central Highlands, Northern Slopes), land‐use determines what species are feeding on (Figure 3b,d). In other bioregions, dietary taxa appeared to be more species‐specific (i.e. Ben Lomond, King, South East; Figure 3a,c,e). The effects of land‐use on dietary diversity changed with bioregions and the species assessed (Table 1). For example, the dietary diversity in Ben Lomond was higher for eastern and spotted‐tailed quolls inhabiting agricultural than plantation landscapes. A similar pattern is observed in Tasmanian devils inhabiting the Central Highlands between agricultural and undisturbed land. However, land‐use did not appear to change the dietary diversity of carnivores on the Northern Slopes. We observed the opposite patterns for swamp rats in different bioregions. In the Northern Slopes, their dietary diversity declines from undisturbed to plantation to agricultural landscapes, while in King, the dietary diversity of swamp rats in plantations is considerably higher than the one in undisturbed landscapes (Table 1). Interestingly, we found that feral cats generally had a more diverse diet than spotted‐tailed quolls when living in sympatry (Table 1).

The following description of the results will be organised by bioregion, then by carnivore versus rodent when available within the bioregion and then by land‐use type. Prey taxa are listed in the order of the amount of eDNA found in each pooled sample, but we acknowledge that these are biased towards a recent meal, especially when the number of pooled samples was low. We did not have samples for all species on every land‐use in every bioregion. If rats (Genus *Rattus*, Family Muridae) are not identified at the species level when describing prey taxa, we refer to them at the genus level (see Table S1).

#### 3.1.1 Ben Lomond

**FIGURE 4:**
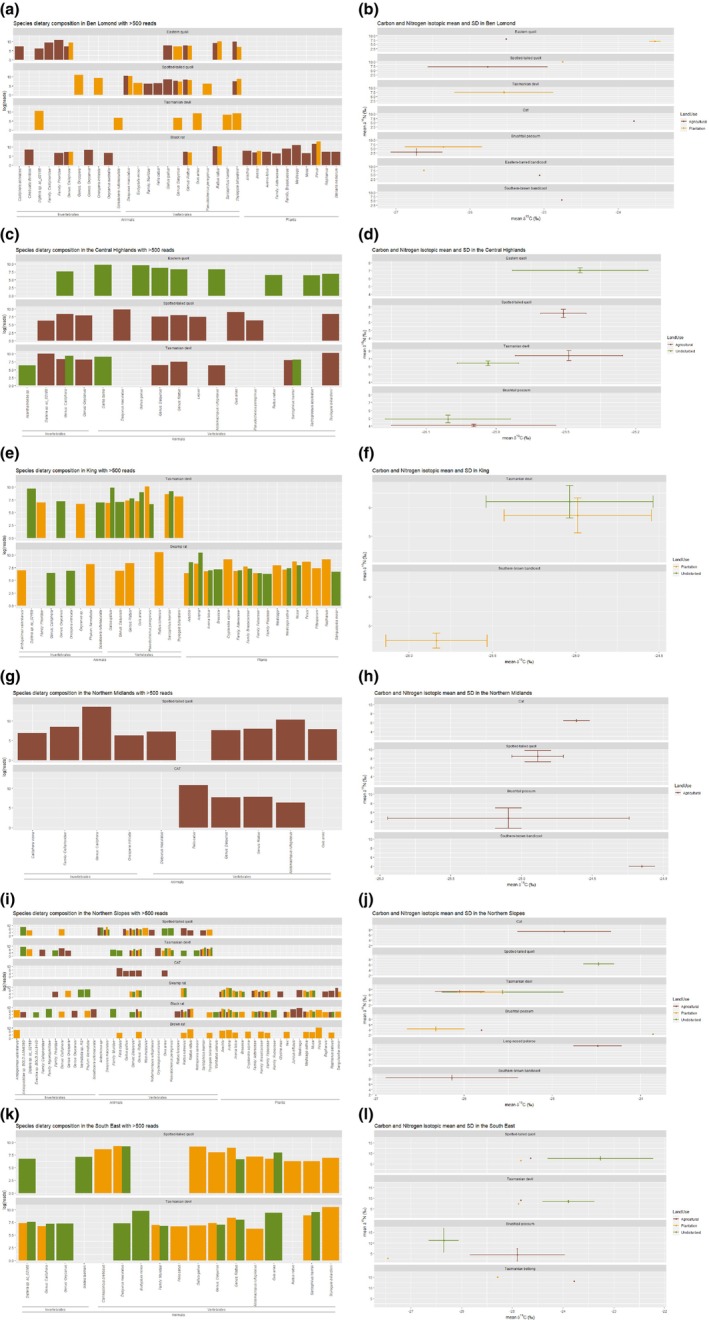
Dietary composition and isotopic niche breadth of native and invasive species per land‐use, per bioregion.

In the Ben Lomond bioregion, we obtained samples for the three native carnivores and the invasive black rat (Figure 4a). No samples were obtained from intact landscapes. In plantations, the main prey item of eastern quolls was black rats, but we also found *Calliphora* flies and pademelons. Spotted‐tailed quolls had fed primarily on moths of the genus *Oncopera*, pademelons and rats, but ringtail possums were also found in their diet. The main prey items found in the faeces of Tasmanian devils in plantation landscapes were pademelons and large quantities of species of the Order Diptera, including *S. rufomaculata*. Our analyses also found significant amounts of quoll (genu*s Dasyurus*) DNA in devil faeces obtained from plantations, which could represent scavenging or predation. In agricultural lands, rats and pademelons were important prey items for both quoll species. However, flies of the Phoridae and Calliphoridae families, both associated with decaying organic matter (animal and plant‐based), were only detected in eastern quolls. We also found the presence of cat DNA in faecal samples of spotted‐tailed quolls in agricultural landscapes.

The diet of black rats trapped in plantations was comprised primarily of pine, followed by flies of the genus *Calliphora*. In agricultural land, pine was also the primary item found in the diet of black rats, but they also fed on plants of the Family Brassicaceae, wood stitchwort (*Stellaria nemorum*) and moths from the genus *Oxycanus* and *Chabuata*.

#### 3.1.2 Central Highlands

In the Central Highlands, samples were obtained only for three native carnivores: eastern and spotted‐tailed quolls and Tasmanian devils (Figure 4c). In the undisturbed landscape of the Central Highlands, fallow deer (*Dama dama*) dominated the diet of the two carnivores for which samples were obtained: eastern quolls and Tasmanian devils. The accompanying large quantities of blowfly (Genus C*alliphora*) reads in both species suggest that these carnivores are scavenging opportunistically on deer carcasses. Bennett's wallaby (*Notamacropus rufogriseus*) and Tasmanian pademelons (*Thylogale billardierii*) found in the diet of eastern quolls are also likely scavenged, although black rats are probably preyed upon.

In agricultural landscapes in the Central Highlands, the main species that characterised the diet of Tasmanian devils and spotted‐tailed quolls was pademelon. Many pademelons in the diet of devils were scavenged, as suggested by the high number of reads assigned to blowflies (*Calliphora* sp.) and other flies (possibly in the form of maggots). Rats and moths from the genus *Oxycarus* were present in both the devil and spotted‐tailed quoll diets. Hares (*Lepus* sp.) were only found in the diet of spotted‐tailed quolls, and Bennett's wallaby was present only in the diet of Tasmanian devils. Sheep were detected in the diet of spotted‐tailed quolls, but this was probably from the bait, as traps were frequently found frozen open with the bait gone.

#### 3.1.3 King

Samples were obtained for just two native species in the King bioregion, Tasmanian devils and swamp rats, and only from intact and plantation landscapes (Figure 4e). The diet of Tasmanian devils in undisturbed landscapes was dominated by DNA reads of rats, several fly species, *Oxycanus* moths and quolls (genus *Dasyurus*). In plantations, DNA reads were abundant for ringtail possums, followed by pademelons and rats, in conjunction with flies and *Oxycanus* moths.

In undisturbed landscapes, the DNA reads of the faeces of swamp rats showed an abundance of medick and other plants of the Musaceae, Brassicaceae and Asteraceae families. In plantations, Brassicaceae and Musaceae were still present in their diet in combination with the prostrate shrub *Cryptandra alpina*, pine and nematodes.

#### 3.1.4 Northern Midlands

Faecal samples for the northern Midlands were obtained only from two carnivores: the native spotted‐tailed quoll and the invasive feral cat, and only from agricultural landscapes, the dominant land‐use in the northern Midlands (Figure 4g). The diet of both carnivore species was dominated by Bennett's wallaby and rats. We also identified flies of the genus *Calliphora* in the samples of spotted‐tailed quolls, indicative of scavenging, probably of wallaby.

#### 3.1.5 Northern slopes

In the Northern Slopes bioregion, samples were obtained for two native (spotted‐tailed quoll, devil) and one invasive (feral cat) carnivore, and one native (swamp rat) and two invasive (black, brown) rats. Cats were only trapped in agricultural landscapes (Figure 4i). Spotted‐tailed quolls and Tasmanian devils both had a high content of common ringtail possums (*Pseudocheirus peregrinus*), rats and Anisopodidae flies in their diets in undisturbed landscapes. *Antechinus* sp. were an important component of the diet of spotted‐tailed quolls. Tasmanian devils also consumed other rodents in the Family Muridae (DNA identified only to family and could be native or invasive rodent), as well as Tasmanian pademelons and Australian smelt (*Retropinna semoni*), a small freshwater fish. In plantations, the diet of spotted‐tailed quolls was dominated by house mice (*Mus musculus*), rats and scavenged pademelons, as suggested again by the number of reads corresponding to *Calliphora* sp. flies. The Tasmanian devil diet included ringtail possums, in addition to pademelons, rats and several species of the order Diptera. The diet of spotted‐tailed quolls in agricultural landscapes was dominated by *Antechinus* sp., followed by native and invasive rats and Bennett's wallaby. Tasmanian devils had high detections of European rabbits (*Oryctolagus cuniculus*), pademelons, flies from the Calliphoridae family and moths from the genus *Oncopera*. Our results also show the presence of quoll (Genus *Dasyurus*) in the diet of Tasmanian devils. Cats relied heavily on rats and also appeared to consume quolls.

In undisturbed areas, the diet of the native swamp rat was dominated by nematodes but included plants in the Genus *Musa* and the Family Brassicaceae. Conversely, the diet of the invasive black rat primarily included plants from the Rubiaceae family and a mix of fly taxa in the Family Mycetophilidae. The Mycetophilidae family, commonly known as fungus gnats, have larval stages associated with the fruiting bodies of fungi, and adult flies can occur in very large congregations among shaded foliage (Colless, 2007). In plantations, the diet of both invasive rat species captured (black rats and brown rats, *R. norvegicus*) was dominated by pine (*Pinus* sp.), followed by the terrestrial slug *Ambigolimax valentianus* and plants of the Brassicaceae family. Additionally, black rats fed on nematodes, salad burnet (*Sanguisorba minor*) and flies (possibly in their larval stage) from the genus *Calliphora*. Swamp rats in plantations were fed primarily on plants in the Families Asteraceae and Brassicaceae but not on nematodes. In agricultural landscapes, swamp rats are fed primarily on salad burnet, followed by medick (*Medicago* sp.), radishes (*Raphanus* sp.) and soybean (*Glycine max*). Medick was also one of the main components in the diet of black rats, alongside bog rush *Juncus effussus*, the fly *Sciadocera rufomaculata* (possibly in larval stages from carcasses), as well as flies from the Anisopodidae family. Swamp rats were found in the diet of black rats in undisturbed and agricultural landscapes, but not the reverse.

#### 3.1.6 South East

In the South East bioregion, samples were obtained only for two native carnivores: the spotted‐tailed quoll and Tasmanian devil, and only from intact and plantation landscapes (Figure 4k). In undisturbed landscapes, the DNA reads of spotted‐tailed quoll faeces were primarily from rats. Ticks (*Ixodes tasmani*) and flies (Diptera) were also present in their scats. The diet of Tasmanian devils in undisturbed landscapes was dominated by little penguins (*Eudyptula minor*), rats and quolls. *Calliphora* flies and *Oxycanus* moths were both detected in the diet of devils. In plantations, spotted‐tailed quolls consumed many rats and also fed on skinks (*Carinascinus pretiosus*), Bennett's wallaby and pademelons. The diet of devils in plantations shifted to pademelons, rats and other rodents, but also included quolls and different species of the Order Diptera.

### 3.2 Stable isotopes

Across all study sites, δ^13^C values ranged from −26.7‰ to −21.9‰ in carnivores and between −27.4‰ and −23.8‰ in omnivores, while δ^15^N values ranged from 5.5‰ to 10.0‰ in carnivores and from 0.91‰ to 15.3‰ in omnivores. Mean δ^13^C but not δ^15^N values appeared to be more similar within bioregions for all carnivores and the herbivorous‐omnivorous brushtail possums (Table 1).

Feral cats usually had less negative δ^13^C values than other sympatric carnivores. The δ^13^C signature of spotted‐tailed quolls tended to be less negative in undisturbed areas and less negative than the δ^13^C signature of Tasmanian devils. Tasmanian devils generally showed similar δ^13^C signatures across land‐uses, especially in wetter habitats (King, Northern Slopes). In drier bioregions (South East, Central Highlands), devils inhabiting undisturbed areas tended to have a different δ^13^C signature than those from managed landscapes (Table 1).

The δ^13^C signatures of brushtail possums did not show much variation with the exception of two agricultural sites (South East and Central Highlands, Table 1), which had values that were distinctively less negative and highly variable. We did not observe any general patterns in the δ^13^C signatures of the peramelids or potoroids: the two bandicoot species, bettongs and potoroos.

Feral cats presented lower δ^15^N values than spotted‐tailed quolls, which in turn followed a pattern across land‐use similar to Tasmanian devils but with lower δ^15^N values. The δ^15^N signature of Tasmanian devils generally varied with land‐use, but there was no consistent pattern across bioregions (Figure 4b,d,f,j,l).

Brushtail possums had δ^15^N values that were relatively uniform across all agricultural landscapes, but that differed from other land‐uses in each bioregion in no specific pattern. Bettongs, potoroos and both species of bandicoots usually had the highest δ^15^N signatures when compared to the δ^15^N values of sympatric species.

## 4 DISCUSSION

This study uses a combination of two complementary methods, eDNA and SIA, to investigate the impact of anthropogenic land‐use on the diet and trophic interactions of multiple species in an invaded community. Our eDNA results show regionalisation in the diet of smaller species (rodents, brushtail possums and eastern quolls), and that within each region, land‐use further determines what species are eating. Regional specialisation in diet was not evident for the two larger predators, the Tasmanian devil and the spotted‐tailed quoll. However, both eDNA and SIA suggest that land‐use did influence the diet of these native carnivores. More specifically, SIA signatures suggest that bioregion and land‐use influence the δ^13^C values of all carnivore species and brushtail possums, whereas only land‐use and not bioregion influence the δ^15^N values of carnivores and possums.

Contrary to our expectations, we did not find patterns that suggest a greater overlap in the diet of native and invasive species in modified landscapes. Across bioregions, native carnivore species generally appear to hunt for available prey but are also highly opportunistic scavengers, as indicated by the detection of saprophagous flies. Invasive feral cats also consume a range of prey, but in our limited samples, we found no evidence of carrion‐associated invertebrates, suggesting they consume either hunted prey or prefer relatively fresh carrion (because fieldwork was carried out during winter, it takes longer for fly larvae to appear on carrion). One limitation of both methods used in this study is that neither can discern between preyed‐on and scavenged food items (Foltan et al., 2005). However, the common detection of saprophagous flies (i.e. Families Calliphoridae and Anisopodidae), likely in the form of maggots, in the eDNA analysis of native carnivores points towards scavenging behaviour. Tasmanian pademelons and Bennett's wallabies are often culled in agricultural landscapes as they compete directly with livestock for grass (Coleman et al., 1997). Moreover, Tasmanian pademelons are among the most abundant roadkill species in Tasmania (Hobday & Minstrell, 2008; Nguyen et al., 2019), providing abundant scavenging opportunities across bioregions. Scavenging appears to be a major source of food for eastern quolls and Tasmanian devils in undisturbed landscapes of the Central Highlands, at least during the winter of this trapping project, where active management of fallow deer (i.e. culling) occurs. Moths of the Hepialidae family (*Oncopera* sp. and *Oxycanus* sp.) were also common items in the diet of spotted‐tailed quolls and Tasmanian devils across all bioregions and land‐use. These are sizeable moths, with various species common in forests and grasslands, and were likely caterpillars during our winter study period (Madge, 1954). Rats (*Rattus* spp.) were a common food item for all carnivore species, native and invasive.

Some food items were associated only with specific regions. This is the case of little penguins and skinks in the diet of Tasmanian devils in the South East and *Antechinus* spp. in the diet of spotted‐tailed quolls in the Northern Slopes. Regional variation in some food items could be related to predator preferences but also to prey abundance and availability. For example, independent studies in snow leopards (Lyngdoh et al., 2014) and black harriers (Garcia‐Heras et al., 2017) showed that these predators presented higher dietary specialisation if their preferred prey was available, while switching to alternative prey otherwise. Little penguins breed in colonies and so constitute an easy source of prey for Tasmanian devils, suggesting opportunistic behaviour. Skink species are widely distributed across Tasmania and are found occasionally in the diet of devils (Jones & Barmuta, 1998), but are so small relative to devil body size that their consumption can be considered opportunistic. Similarly, the diet of spotted‐tailed quolls usually contains large proportions of rats, but not in the Northern Slopes, despite this bioregion having the highest rates of rat captures in this study. The otherwise similar similarities in diet among the sympatric native carnivores suggest that for these species, diet is related to prey abundance and availability, and that Tasmanian devils and both species of quolls are opportunistic hunters and scavengers, which agrees with previous research (Glen & Dickman, 2006; McLennan et al., 2022; Pemberton et al., 2008).

The opportunistic nature of foraging in devils and quolls is further supported by the additional influence of land‐use on the diet composition of these species. For example, ringtail possums were found almost exclusively in diet samples of Tasmanian devils and spotted‐tailed quolls inhabiting plantations. Likewise, rabbits and hares were only found in the diets of carnivores inhabiting agricultural land. This is most likely explained by the abundance of ringtail possums and lagomorphs in plantations and agricultural landscapes, respectively (Hamer et al., 2021; Hollings et al., 2014). The degree of variation in the Simpson index of diversity among land‐uses within each bioregion also points towards devils and spotted‐tailed quolls feeding on available food items because these variations do not follow a pattern across species, land‐use or bioregion. Hence, our results suggest that any degree of trophic specialisation (Lewis et al., 2022) in these species within each landscape is dependent on the presence and abundance of available food items (e.g. McLennan et al., 2022) and sympatric functional groups, namely, that context matters.

Some degree of trophic niche partitioning is evident among the native carnivores and between them and the invasive feral cat. In agricultural landscapes, for which feral cat samples were available, δ^13^C values show small increases from devils to spotted‐tailed quolls to feral cats, while δ^15^N values follow the opposite direction. Our eDNA analysis shows that cats feed primarily on rats, whereas spotted‐tailed quolls and devils consume rats, moths and a variety of carrion that was likely infected with the larval stages of several fly species. In the Northern Midlands, however, where Tasmanian devils are present only in very low numbers and no diet samples were obtained, results of eDNA analyses show Bennett's wallaby in the samples obtained from feral cats. Previous observations suggest that the decline in Tasmanian devils, morphologically specialised scavengers that account for most of the consumption of carrion in Tasmania, has provided feral cats and other scavengers with a feast of unconsumed carrion (Cunningham et al., 2018). However, the absence of saprophagous flies in the eDNA samples of feral cats suggests that feral cats are consuming carrion before these flies lay their eggs in it, which takes longer in winter. This highlights the dynamic nature of invaded ecosystems and emphasises the need for any hint of trophic specialisation to be analysed in different contexts.

Our analyses also show trophic segregation among the native carnivore species and that such segregation probably occurs at the level of hunted prey species. This segregation reflects the body size and foraging mode of each species relative to the prey. For example, in undisturbed landscapes in the Central Highlands, eastern quolls and devils fed on deer carcasses, which are large and would have been scavenged, but eastern quolls also fed on rats, which they probably killed. In agricultural land, spotted‐tailed quolls and devils both fed on pademelons, which they are both capable of killing, but only the extremely agile spotted‐tailed quolls fed on hares. In undisturbed areas in the South East, devils and spotted‐tailed quolls fed on rats, but little penguins were only found in the diet of devils. Devils are capable diggers and can decimate seabird colonies by digging both adults and chicks out of breeding burrows (Scoleri et al., 2020). Finally, in plantation landscapes in Ben Lomond, while eastern quolls fed primarily on black rats and scavenged on pademelons, spotted‐tailed quolls fed on rats and pademelons but also hunted moths and ringtail possums, and devils relied primarily on pademelons. Our SIA further supported fine‐scale niche segregation at the larger time scales represented by this analytical approach. Segregation on hunted prey taxa, but convergence on scavenged prey, makes sense. Mammalian predators have trophic and skeletal morphological specialisations that adapt them to hunting and killing particular types of prey (Jones, 2003; Van Valkenburgh, 1987, 1989). Scavenging per se requires fewer morphological adaptations, although a few species like Tasmanian devils and hyaenas have specialised dentition and skull morphology for osteophagy or bone‐eating (Jones, 2003; Van Valkenburgh, 1987, 1989), and smaller species may rely on commensal relationships with larger scavengers to access tough carcasses (e.g. eastern quolls rely on devils; Jones, 1995). Otherwise, carrion‐feeding provides a nutritional food resource with little energetic cost of foraging, although there is some risk of interference with competition (Jones, 1995).

We also observed trophic niche partitioning between native and invasive rat species and changes in their diet with land‐use. Managed landscapes have a strong effect on the diversity of food items ingested by the native swamp rat when compared to undisturbed areas. This was generally not the case for black rats. The trophic niche partitioning we observed between native and invasive rats might suggest that coexistence between these species is possible. The diet of swamp rats is omnivorous and is typically associated with an abundance of stem and leaf material, with some presence of roots, insects and fungi (Driessen, 1987; Watts & Braithwaite, 1978). Black rats are also omnivores but are more carnivorous than the native rat and have a broader trophic niche (Clark, 1982). Our results agree with these findings. We also found black rats to prey on the native swamp rat but not the reverse, reflecting their different trophic envelopes and highlighting the conservation risk for native rodents from the invasive black rat. Outstanding questions are whether and how black rats influence diet modification in native swamp rats when they live in sympatry, and the potential long‐term consequences of a flexible trophic niche in the native swamp rat for its conservation (e.g. Birnie‐Gauvin et al., 2017). Of potential concern is competition for food resources. For example, we found large quantities of nematodes in the diet of swamp rats where black rats were not trapped (despite trapping effort being equal at all sites, suggesting the possibility of a smaller population of black rats). Yet, in landscapes where both species were captured, nematode reads were absent in the diet of swamp rats but present in large quantities in black rat faeces. Given that we only obtained taxonomic resolution for nematodes at the phylum level, it is possible that native and invasive rat species have preferences for different free‐living nematodes, and that the dietary shift is rather a result of land‐use impacting the availability of specific nematode species. We believe these nematodes to be free‐living nematodes because they were only found in the above‐mentioned samples, and had they been parasitic, we would have expected to find reads in all wildlife samples (e.g. Spratt & Beveridge, 2016).

Although we did not have eDNA data for brushtail possums, the δ^15^N values suggest that land‐use also influences their diet. The variation in δ^15^N values among landscapes could be related to changes in the proportion of eggs and insects consumed by possums, with higher δ^15^N values associated with a higher consumption of these items (Cruz et al., 2012; Foulkes, 2001). Interestingly, all δ^15^N values in agricultural landscapes overlap, suggesting that possums might be supplementing their diet by grazing and feeding on crops (Smith, 2012), which would be relatively similar across bioregions. Brushtail possums are arboreal but descend to the ground to graze on grass and crops wherever predation risk from Tasmanian devils is low (Cunningham et al., 2019; Hollings et al., 2015), probably because these plants have lower anti‐browsing chemicals than tree leaves (Lawler et al., 1998, 2000). Grazing would also explain the larger variation found in δ^13^C signatures of brushtail possums in agricultural sites because grasses, where C4 photosynthesis is most prevalent (Sage et al., 1999), will have less negative δ^13^C values (Tieszen & Boutton, 1989).

Finally, we also observed the highest δ^15^N values in both bandicoot species, bettongs and potoroos when compared to sympatric species sampled, and δ^13^C values within the average range observed for all other sympatric species. Our small sample size and lack of captures in certain landscapes hinder our study from assessing the effects of land‐use on the diet of bandicoots, bettongs and potoroos. The higher δ^15^N values found for these species are in line with the isotopic values found for other bandicoot and bettong species in Australia (McIlwee & Johnson, 1998). McIlwee and Johnson (1998) found high δ^15^N values to be indicative of the consumption of large amounts of fungi. This would also explain the elevated δ^15^N values in long‐nosed potoroos, which rely primarily on fungi for their diet (Tory et al., 1997). Southern brown and eastern barred bandicoots feed primarily on invertebrates (earthworms, slugs and larval stages of insects) and occasionally on berries when available (Heinsohn, 1966), but southern brown bandicoots have also been recorded feeding on grasses, fungi and seeds (Driessen & Rose, 2015). Because insects also occupy different trophic levels, large differences are observed in the δ^15^N signature of different insect families and orders (Benneth & Hobson, 2009). Consequently, insectivorous species will on average show higher δ^15^N values than their herbivorous counterparts (e.g. Balčiauskas et al., 2019).

Changes in land‐use across the world are shown to influence the diet of local wildlife, both carnivores and herbivores, suggesting that species adapt their diet to available resources. Among predators, human land‐use provides subsidies for introduced prey and modifies the availability of native prey. For example, Farias and Kittlein (2008) showed that predatory pampas foxes (*Pseudalopex gymnocercus*) in the Argentinian Patagonia cope with habitat alteration by subsidising their diet with highly available introduced species (i.e. European hare, *Calomys* rodents and ungulate carrion). Abbas et al. (2011) also found dietary plasticity in the herbivorous European roe deer (*Capreolus capreolus*) in habitats with different degrees of fragmentation. Wang et al. (2021), on the other hand, found that potential competitive outcomes between native and invasive macroinvertebrates, resulting from habitat alteration, rely on the diversity and availability of prey. In all cases, it appears that species adapt their diet to the availability of prey, and this also appears to be the case in our study system. Although diet modification reduces interspecific competition and potentially allows for species coexistence and adaptation to altered habitats, it raises several other questions. For example, dietary shifts can lead to the consumption of food items of lower quality, with potential long‐term impacts on life‐history traits and evolutionary changes that are not yet clearly understood (Birnie‐Gauvin et al., 2017; Roff, 2002; Shik & Dussutour, 2020). At the opposite end, what are the potential impacts on behaviour and population dynamics of the new exploited resources? Are we witnessing early stages of dietary shifts and adaptation? Are species in these ‘transitioning periods’ more vulnerable to further changes in their environment? What do dietary adjustments mean to native biodiversity?

Our study demonstrates that human activities are moulding the trophic relationships of this invaded community, not only at the level of land‐use, but also by subsidising carrion through the provision of large carcasses from animal management and roadkill (Coleman et al., 1997; Hobday & Minstrell, 2008; Nguyen et al., 2019). In Tasmania, the impact of carrion availability on mammal and avian species communities is exacerbated by the massive disease‐caused decline in Tasmanian devils (Cunningham et al., 2018; Fielding et al., 2021), the top predator and scavenger in this ecosystem, which is causing trophic changes cascading through both the consumptive and detritivorous parts of the food web (Cunningham et al., 2020). We acknowledge the several shortcomings in our study, some of which are inherent to the type of analyses we used, such as the dependency on the availability of assigned sequences in public databases for taxa detection (Kwong et al., 2012) and the constrains of the selected primers and their associated biases (Elbrecht & Leese, 2015). Additional constraints included sample size and particularly, being able to obtain samples of all species at each site. Such limitations can be overcome in future studies by increasing sampling effort and seasonal changes. Our study does provide the first insights into the trophic dynamics of an invaded system under different anthropogenic contexts. Our results stress just how important it is to include human activities in ecological studies. Our study highlights the value of using two complementary methods to study trophic dynamics and to simultaneously study multiple species in a community to gain a better understanding of the synergetic impacts on native biodiversity.

## AUTHOR CONTRIBUTIONS

**Antje Chiu‐Werner:** Conceptualization (equal); formal analysis (lead); funding acquisition (equal); investigation (lead); methodology (lead); writing – original draft (lead); writing – review and editing (equal). **Menna Jones:** Conceptualization (equal); formal analysis (supporting); funding acquisition (equal); investigation (supporting); methodology (supporting); supervision (lead); writing – original draft (supporting); writing – review and editing (equal).

## CONFLICT OF INTEREST STATEMENT

The authors declare no conflict of interest.

### OPEN RESEARCH BADGES

This article has earned an Open Data badge for making publicly available the digitally‐shareable data necessary to reproduce the reported results. The data is available at https://doi.org/10.6084/m9.figshare.22290829.

## Supporting information


Table S1

## Data Availability

The original datasets containing the carbon and nitrogen isotopic signatures, and the diet components for each species per land‐use will be made available via figshare. Data are provided for peer review at the following link: https://figshare.com/s/9d44156bcbc303369755.
