# Supplementary material for: Human land‐use changes the diets of sympatric native and invasive mammal species
Source: Ecol Evol. 2023 Dec 6;13(12):e10800. doi: 10.1002/ece3.10800 (PMC10700046; doi:10.1002/ece3.10800)
Supplement: Supplementary file 1 — Table S1 [file ECE3-13-e10800-s001.docx]

Supplementary Table 1. Total number of dietary items per species per bioregion per land-use as found by eDNA analysis

| **Species** | **Bioregion** | **Land Use** | **Number of different dietary items** |
| --- | --- | --- | --- |
| Black rat | Ben Lomond | Agricultural | 45 |
| Black rat | Ben Lomond | Plantation | 39 |
| Black rat | Northern Slopes | Agricultural | 41 |
| Black rat | Northern Slopes | Undisturbed | 47 |
| Black rat | Northern Slopes | Plantation | 42 |
| Brown rat | Northern Slopes | Plantation | 34 |
| Cat | Northern Slopes | Agricultural | 23 |
| Cat | Northern Midlands | Agricultural | 18 |
| Eastern quoll | Ben Lomond | Agricultural | 26 |
| Eastern quoll | Ben Lomond | Plantation | 22 |
| Eastern quoll | Central Highlands | Undisturbed | 24 |
| Swamp rat | King | Undisturbed | 29 |
| Swamp rat | King | Plantation | 40 |
| Swamp rat | Northern Slopes | Agricultural | 28 |
| Swamp rat | Northern Slopes | Undisturbed | 38 |
| Swamp rat | Northern Slopes | Plantation | 45 |
| Spotted-tailed quoll | Ben Lomond | Agricultural | 15 |
| Spotted-tailed quoll | Ben Lomond | Plantation | 24 |
| Spotted-tailed quoll | Central Highlands | Agricultural | 23 |
| Spotted-tailed quoll | Northern Midlands | Agricultural | 29 |
| Spotted-tailed quoll | Northern Slopes | Agricultural | 20 |
| Spotted-tailed quoll | Northern Slopes | Undisturbed | 22 |
| Spotted-tailed quoll | Northern Slopes | Plantation | 25 |
| Spotted-tailed quoll | South East | Undisturbed | 22 |
| Spotted-tailed quoll | South East | Plantation | 25 |
| Tasmanian devil | Ben Lomond | Plantation | 25 |
| Tasmanian devil | Central Highlands | Agricultural | 26 |
| Tasmanian devil | Central Highlands | Undisturbed | 22 |
| Tasmanian devil | King | Undisturbed | 28 |
| Tasmanian devil | King | Plantation | 23 |
| Tasmanian devil | Northern Slopes | Agricultural | 22 |
| Tasmanian devil | Northern Slopes | Undisturbed | 26 |
| Tasmanian devil | Northern Slopes | Plantation | 24 |
| Tasmanian devil | South East | Undisturbed | 22 |
| Tasmanian devil | South East | Plantation | 24 |
